# Dynamic Vessel Analyzer (DVA) – a new method to detect endothelial dysfunction in chronic heart failure: correlation between DVA and asymmetric dimethyl arginine (ADMA)

**DOI:** 10.1186/1471-2210-11-S1-P66

**Published:** 2011-08-01

**Authors:** Markus Schwefer, Aleksandra Mandecka, Helen Schmicker-Helf, Andreas Brückmann, Thomas Lehmann, Ingeburg Schauer, Frank Kramer, Marcus Blum, Harald Lapp

**Affiliations:** 1 Helios Klinikum Erfurt, Department of Cardiology, Nordhaeuser Strasse 74, D-99089 Erfurt, Germany; 2 University of Jena, Department of Internal Medicine III, Erlanger Allee 101, D-07740 Jena, Germany; 3 University of Jena, Department of Obstetrics, Bach Strasse 18, D-07743 Jena, Germany; 4 University of Jena, Institute of Medical Statistics, Information Sciences and Documentation, BachStrasse 18, D-07743 Jena, Germany; 5 Helios Klinikum Erfurt, Dia-Solution GmbH, Nordhaeuser Strasse 74, D-99089 Erfurt, Germany; 6 Bayer Schering Pharma AG, Global Biomarker Research, Pharma Research Center, Aprather Weg 18a, D-42096 Wuppertal, Germany; 7 Helios Klinikum Erfurt, Department of Ophthalmology, Nordhaeuser Strasse 74, D-99089 Erfurt,Germany; 8 University of Witten Herdecke, Medical Faculty, Alfred-Herrhausen-Strasse 50, D-58448 Witten Herdecke, Germany

## Background

Chronic heart failure (CHF) is the leading cause of hospitalization and death in industrialized countries. CHF is frequently associated with humoral and metabolic disturbances, including reduced bioavailability of the important signalling molecule nitric oxide (NO), which has vasodilating properties. Several studies reported high plasma levels of asymmetrical NG, NG-dimethyl-L-arginine (ADMA), an endogenous inhibitor of NO production, in CHF, contributing to endothelial dysfunction. The Dynamic Vessel Analyzer (DVA) enables dynamic analyses of retinal vessels. NO is a mediator of retinal vasodilator response to flicker light. Reduced response of retinal arterioles to flicker light may be an attractive technique to non-invasively assess endothelial dysfunction. The aim of the study was to test the hypothesis that retinal vessel response to flicker light is reduced in patients with CHF and correlates inversely with serum ADMA levels.

## Methods and results

16 patients with non-ischemic cardiomyopathy and 22 healthy volunteers were included. Retinal arteriolar flicker response as percent change from baseline and serum ADMA level were measured.

Retinal arteriolar flicker response was significantly reduced in CHF patients compared to the healthy control group (Median: 0.60 vs. 4.60%; p<0.001). ADMA levels tended to be higher in CHF patients (median: 0.66 vs. 0.62 μmol/L; p=0.099). Noteworthy, we observed a highly significant inverse correlation between retinal arteriolar flicker response and ADMA levels (r = -0.531, p=0.001).

## Conclusions

Our findings suggest that analysis of retinal vessels could be an attractive non-invasive method to quantify endothelial dysfunction in CHF.

